# *ALDH2* rs671 variant allele is associated with higher energy intake in middle-aged and elderly Japanese who routinely consume alcohol

**DOI:** 10.1265/ehpm.22-00276

**Published:** 2023-05-11

**Authors:** Hiroyuki Hayashida, Akiko Matsumoto, Hinako Nanri, Yuichiro Nishida, Yusuke Takagi, Megumi Hara

**Affiliations:** 1 Faculty of Medicine, Saga University, 5-1-1 Nabeshima, Saga 849-8501, Japan; 2 Department of Environmental and Social Medicine, Saga University, 5-1-1 Nabeshima, Saga 849-8501, Japan; 3 Department of Physical Activity Research, National Institutes of Biomedical Innovation, Health and Nutrition, 1-23-1 Toyama, Shinjuku, Tokyo 162-8636, Japan; 4 Laboratory of Gut Microbiome for Health, Collaborative Research Center for Health and Medicine, National Institutes of Biomedical Innovation, Health and Nutrition, 7-6-8 Saito-Asagi, Ibaraki, Osaka 567-0085, Japan; 5 Department of Preventive Medicine, Faculty of Medicine, Saga University, 5-1-1 Nabeshima, Saga 849-8501, Japan

**Keywords:** ALDH2, Aldehyde dehydrogenase, Alcohol, Energy intake, Macronutrient intake

## Abstract

**Background:**

According to recent reports, individuals with reduced aldehyde dehydrogenase activity may require more energy for the detoxification of aldehydes. Aldehyde dehydrogenase 2 (ALDH2), an ALDH isozyme, is responsible for detoxifying acetaldehyde, an intermediate metabolite of ethanol. Because the variant allele of the rs671 polymorphism of *ALDH2* results in a substantial reduction in enzymatic activity, carriers of this variant allele may have a higher energy demand when consuming alcohol than non-carriers. However, no studies have evaluated this phenomenon to date.

**Method:**

To test the hypothesis, we statistically examined the interactive effects between the rs671 and ethanol consumption on energy intake using cross-sectional data from a population-based cohort study, the Japan Multi-Institutional Collaborative Cohort Study, which was conducted in Saga city between 2005–2007 (N = 12,068).

**Results:**

General linear regression models adjusted for age, sex, ethanol consumption, current smoking status, years of education, dietary restriction, medical history, and physical activity level revealed that energy intake was higher in variant allele carriers than in non-carriers among individuals with alcohol drinking habits, whereas no such correlation was observed among those without drinking habits (≤2 g ethanol/day) (*p* = 0.03 for interaction between rs671 and ethanol consumption). Energy intake excluding energy from alcoholic beverages, carbohydrate intake, protein intake, and fat intake, showed similar tendencies (*p* for interaction = 0.01, 0.01, 0.04, and 0.07, respectively).

**Conclusions:**

These findings support the hypothesis that increased energy intake is required for the detoxification of aldehydes in individuals with low ALDH activity. This epidemiological evidence provides a possible scientific basis for understanding aldehyde detoxification mechanisms and suggests a novel phenotype of the *ALDH2* rs671 polymorphism.

**Supplementary information:**

The online version contains supplementary material available at https://doi.org/10.1265/ehpm.22-00276.

## 1. Introduction

Alcohols are metabolized by alcohol dehydrogenase to aldehydes, which is in turn metabolized by aldehyde dehydrogenase (ALDH) to carboxylic acid. ALDH2, an ALDH isozyme, has the highest affinity for acetaldehyde and is responsible for its detoxification [1]. Although there are numerous polymorphisms of *ALDH2*, only the rs671 has a major effect on ALDH2 enzymatic activity [2, 3]. Most of the global population has the wild-type allele, homozygous ALDH2*1 (ALDH2*1/*1). However, in the East Asian region, many people carry the variant allele ALDH2*2, or genotype of ALDH2*1/*2 or ALDH2*2/*2. The ALDH2*2 carriers reportedly exceed 60% in a particular area in southeastern China [4], are relatively common in Japan and Taiwan (approx. half of the population) [4–6], and less common in Korea and northern China (10–40%) [4, 7, 8]. ALDH2*2 is associated with various kinds of phenotypes [9–14], including disease susceptibility [e.g., high-risks of alcohol-related cancer among alcohol-users [15], atrial fibrillation among alcohol-users [16], and Alzheimer’s diseases [17]], immunotherapy, and vaccine sensitivity [10, 11]. The best known is the so-called “Asian flush” phenomenon, in which, acetaldehyde accumulates upon alcohol consumption, leading to skin flushing, in some cases over the entire body, nausea, palpitations, drowsiness, and headache [1, 18, 19]. Because of these symptoms, ALDH2*2 carriers generally tend to avoid consuming ethanol [1, 14]. However, many of them do consume alcoholic beverages, and moreover, 10–20% of patients with alcohol use disorder are carriers of ALDH2*2 [13, 20, 21].

Naturally, habitual drinking in ALDH2*2 carriers is a great risk for alcohol-related cancers, such as esophageal cancer, because accumulated acetaldehyde is highly carcinogenic [20, 22–25]. Nevertheless, drinking habits of ALDH2*2 carriers may have been more easily overlooked by medical professionals, because indicators of overdrinking such as serum liver transaminase levels, blood pressure, serum triglyceride levels, and visceral fat content, are reported to be lower in ALDH2*2 carriers [20, 26–36] even with consideration of drinking amount [26, 28, 35, 36]. The mechanisms underlying this favorable, but enigmatic traits in ALDH2*2 carriers have not been revealed; however, the accumulation of aldehydes is a primary possible reason, considering the role of ALDH2.

Using an animal model, we previously reported unexpected and protective effects of ALDH2 deficiency against ethanol loading [27, 37–39], including higher hepatic glutathione (GSH) and lower lipid peroxide levels in *Aldh2* knockout mice than in wild-type mice after single ethanol administration [37]. Subsequently, Endo et al. [40] reported unexpected tolerance to aldehydes in a rodent model overexpressing inactive ALDH2, exhibiting impaired ALDH activity. They discovered a metabolic remodeling characterized by increased energy consumption, inducing GSH detoxification of aldehydes [40]. This indicated that aldehyde loading is associated with higher energy demands because of a compensatory detoxification system in individuals with reduced ALDH activity. These findings led us to hypothesize that carriers of the *ALDH2* rs671 variant allele (ALDH2*2) who consume alcohol habitually, consume higher amounts of foods to compensate for the increased energy demands for aldehyde detoxification, which possibly explains both the unexpected tolerance to aldehydes and the low risk of drinking-related metabolic disorders, that is, the indicators of overdrinking, in this population.

Using a Japanese population-based cohort database, we aimed to examine our hypothesis epidemiologically. By assessing the interactions between the *ALDH2* rs671 polymorphism and ethanol consumption on energy and macronutrient intake, we tried to clarify if ALDH2*2 allele is associated with higher energy intake among individuals who routinely consume alcohol.

## 2. Methods

### 2.1. J-MICC Saga study

We used baseline survey data from a population-based cohort study, the Japan Multi-Institutional Collaborative Cohort (J-MICC) Study, conducted between 2005–2007 in Saga City, Japan. Among the 61,447 individuals who were invited, 12,068 completed the baseline survey (19.7% participation rate) [41, 42]. Participants included men and women aged between 40–69 years living in former Saga City (currently the center part of Saga City). The J-MICC Study protocol was approved by the ethics committees of the Nagoya University Graduate School of Medicine (project ID: 253) and the Saga University Faculty of Medicine (project ID: H17-11). Written informed consent for genetic analysis and publication was obtained from all participants.

### 2.2. Field survey

All J-MICC Study participants completed a self-administered questionnaire which was used to collect information regarding ethanol consumption, current smoking status, dietary habits, medical history (liver cirrhosis, diabetes mellitus, hyperlipidemia, hypertension, ischemic heart disease, and stroke), years of education (≤9 years, 9–12 years, and >12 years), and dietary restrictions (salt, calories, sugar, and fat). In addition to weight measurements and venous blood sampling, physical activity was measured using a uniaxial accelerometer (Lifecorder; Suzuken Co., Ltd., Nagoya, Japan) worn on the waist for 10 days, except during sleeping and bathing. The physical activity level was calculated as the total energy expenditure divided by the basal metabolic rate as reported elsewhere [43, 44]. DNA extracted from buffy coats of blood samples was stored at −80 °C until analysis. The *ALDH2* rs671 polymorphism was determined using real-time polymerase chain reaction and the TaqMan^®^ SNP Genotyping Assay (Thermo Fisher Scientific, Inc., Waltham, MA, USA) as per the manufacturer’s protocol.

### 2.3. Ethanol consumption

Daily total ethanol consumption was estimated based on the ethanol concentration and consumption frequency (low, 1–3 days/month, 1–2 days/week, 3–4 days/week, 5–6 days/week, daily) of sake, shochu, chuhai, beer, whiskey, and wine. Consumption was standardized to 60 kg body weight (g ethanol/day/60 kg body weight), the average body weight of the cohort, because ethanol diffuses in many tissues including adipose tissue, liver, and brain [45, 46]; therefore, the consumption amount per body weight may be better reflected by the intracellular ethanol concentration. Using the definition of moderate ethanol consumption of the Japanese Ministry of Health, Labour and Welfare, 20 g/day, individuals who routinely consumed alcohol were divided into moderate (≤20 g/day) and heavy drinkers (>20 g/day); those who consumed ≤2 g/day were considered non-drinkers.

### 2.4. Dietary intake

Dietary assessment was conducted as shown in a previous report [41]. Briefly, data were collected using the validated short food frequency questionnaire (FFQ) developed by Tokudome et al. [47, 48]. The FFQ assesses the average intake of 47 food and beverage items over the past year. The reproducibility of the FFQ over four seasons was well evaluated [49] and validity in comparison with 3-day weight diet records were reasonable [48, 49]. Alcoholic beverages are one of the 47 items on the questionnaire; however, instead of using the original FFQ for alcohol beverages, the above method (see 2.2.1 Ethanol Intake) was used to obtain more detailed information. The frequency of the three staple foods (rice, bread, and noodles) was determined by the following categories (frequency per day in parentheses): almost none (0), 1–3/m (0.1), 1–2/w (0.2), 3–4/w (0.5), 5–6/w (0.8), and 7/w (1). The quantity of staple foods was reported as bowls/day for rice and noodles, and as slices (or rolls)/day for bread. For the other 43 dietary items, only frequencies were reported as follows (frequency per day in parentheses): almost none (0), 1–3/m (0.1), 1–2/w (0.2), 3–4/w (0.5), 5–6/w (0.8), 7/w (1), 2/day (2), and ≥3/day (3). The estimated dietary intake from the FFQ was standardized to 60 kg body weight, because the energy requirement for individuals is proportional to body weight.

### 2.5. Exclusion criteria

We excluded 1,010 J-MICC participants with the homozygous genotype ALDH2*2/*2 from the present analysis, because most carriers of ALDH2*2/*2 do not have alcoholic drinking habits [12, 20, 50]. Another 158 participants were excluded for the following reasons: lack of data on body weight (N = 12) or the rs671 genotype (N = 138), total energy intake ≥3,500 or ≤500 kcal/day (N = 11). Thus, data on 10,910 individuals were eventually included in the analysis.

### 2.6. Statistical analysis

The data of 10,910 individuals were subjected to general linear regression analysis with α = 0.05. Outcomes were logarithmically transformed to approximate a normal distribution. All missing data for covariates, such as years of education (N = 30), current smoking status (N = 1), hyperlipidemia (N = 5), hypertension (N = 1), ischemic disease (N = 4), stroke (N = 1), and physical activity level (N = 160), were assumed to have occurred at random. All analyses were performed using SAS 9.4 (SAS Institute Inc., Cary, NC, USA). Statistical methods are shown with figures and tables.

#### 2.6.1. Outcomes, explanatory variables, and covariates

The primary outcomes were 1) total energy intake, 2) energy intake excluding energy from alcoholic beverages, 3) carbohydrate intake, 4) protein intake, and 5) fat intake, and explanatory variables were the rs671 genotype (ALDH2*1/*1 or ALDH2*1/*2) and/or ethanol consumption level (ordinal variable with 0, 1, and 2 for non-drinkers, moderate drinkers, and heavy drinkers, respectively, or continuous variable). Variables considered to be associated with energy intake or the rs671 polymorphism, including age (continuous variable), sex, years of education (categorical variable), current smoking status (categorical variable), medical history (categorical variable), dietary restrictions (categorical variables), and physical activity level (continuous variable), were used as covariates. Ethanol consumption level (continuous variable) was further included as a covariate when the differences in the amounts of ethanol consumption between genotypes needs to be adjusted.

#### 2.6.2. Interactive analysis

To examine the hypothesis that the relationships between ethanol consumption and energy or macronutrient intake differ based on the *ALDH2* genotype, interactive analyses were performed by multivariate linear regression models (model A and B), which include an interactive term of *genotype* × *ethanol consumption* (*continuous variable*). Model A included the *ALDH2* rs671 genotype, ethanol consumption, and interaction term as explanatory variables, with sex and age as covariates. Model B included explanatory variables and covariates in model A and additional covariates of years of education, current smoking status, dietary restrictions, medical history, and physical activity level.

#### 2.6.3. Sensitivity analysis

Sensitivity analyses were performed using body height or body fat percentage as an additional covariate because the rs671 polymorphism may affect physique. Additional analysis with stratification by sex was performed because alcohol consumption was substantially higher in men than in women.

## 3. Results

### 3.1. Study population characteristics

The characteristics of the study subjects are presented in Table 1. There were no differences between *ALDH2* genotypes in terms of age, sex, years of education, body weight, and current smoking status. ALDH2*1/*2 carriers had significantly lower levels of ethanol consumption than ALDH2*1/*1 carriers, and the significance remained after stratification by ethanol consumption level, that is, 0.0 vs. 0.0 g/day in non-drinkers (Table S1), 7.8 vs. 7.7 g/day in moderate drinkers (Table S2), and 40 vs. 36 g/day in heavy drinkers (Table S3). The prevalence rates of diabetes mellitus, hypertension, and stroke, as well as salt intake restriction, were lower in ALDH2*1/*2 than in ALDH2*1/*1 carriers (Table 1). Although no difference in total energy intake was observed between the *ALDH2* genotypes, energy intake excluding energy from alcoholic beverages, carbohydrate intake, protein intake, and fat intake, were higher in ALDH2*1/*2 than in ALDH2*1/*1 carriers (Table 1).

**Table 1:** Characteristics of all participants based on *ALDH2* rs671 polymorphism

	**Total**		ALDH2*1/*1		ALDH2*1/*2		***p-value***
All, n	10910	(100%)	6131	(56.2%)	4779	(43.8%)	
Age, years	57	(49–63)	57	(49–63)	57	(50–63)	*0.9835*
Sex							*0.3422*
Men, n	4572	(41.9%)	2545	(41.5%)	2027	(42.4%)	
Women, n	6338	(58.1%)	3586	(58.5%)	2752	(57.6%)	
Years of education							*0.7879*
≤9 years, n	645	(5.9%)	355	(5.8%)	290	(6.1%)	
9–12 years, n	5422	(49.8%)	3058	(50%)	2364	(49.6%)	
>12 years, n	4813	(44.2%)	2698	(44.2%)	2115	(44.4%)	
Ethanol consumption†							
Median, g/day	3.4	(0–19.3)	7.8	(0–28.8)	0	(0–8.7)	*<0.0001*
≤2 g/day, n	4895	(44.9%)	2030	(33.1%)	2865	(60%)	*<0.0001*
2–20 g/day, n	3352	(30.7%)	2085	(34%)	1267	(26.5%)	
>20 g/day, n	2663	(24.4%)	2016	(32.9%)	647	(13.5%)	
Current smoking							*0.9374*
Yes, n	2179	(20%)	1223	(20%)	956	(20%)	
No, n	8730	(80%)	4908	(80.1%)	3822	(80%)	
BW (kg)	57.4	(50.8–65.8)	57.4	(50.9–65.9)	57.4	(50.6–65.5)	*0.2099*
Medical history							
Liver cirrhosis							*0.2897*
No, n	10868	(99.6%)	6104	(99.6%)	4764	(99.7%)	
Yes, n	42	(0.4%)	27	(0.4%)	15	(0.3%)	
Diabetes mellitus							*0.0322*
No, n	10202	(93.5%)	5705	(93.1%)	4497	(94.1%)	
Yes, n	706	(6.5%)	424	(6.9%)	282	(5.9%)	
Hyperlipidaemia							*0.7504*
No, n	8800	(80.7%)	4950	(80.8%)	3850	(80.6%)	
Yes, n	2105	(19.3%)	1176	(19.2%)	929	(19.4%)	
Hypertension							*<0.0001*
No, n	8773	(80.4%)	4823	(78.7%)	3950	(82.7%)	
Yes, n	2136	(19.6%)	1307	(21.3%)	829	(17.4%)	
Ischemic heart disease							*0.462*
No, n	10583	(97%)	5952	(97.1%)	4631	(96.9%)	
Yes, n	323	(3%)	175	(2.9%)	148	(3.1%)	
Stroke							*0.0386*
No, n	10737	(98.4%)	6020	(98.2%)	4717	(98.7%)	
Yes, n	172	(1.6%)	110	(1.8%)	62	(1.3%)	
Dietary restrictions							
Salt							*0.0157*
No, n	4812	(44.1%)	2642	(43.1%)	2170	(45.4%)	
Yes, n	6098	(55.9%)	3489	(56.9%)	2609	(54.6%)	
Caloric							*0.6343*
No, n	5959	(54.6%)	3361	(54.8%)	2598	(54.4%)	
Yes, n	4951	(45.4%)	2770	(45.2%)	2181	(45.6%)	
Sugar							*0.2112*
No, n	6477	(59.4%)	3608	(58.9%)	2869	(60%)	
Yes, n	4433	(40.6%)	2523	(41.2%)	1910	(40%)	
Fat							*0.514*
No, n	5025	(46.1%)	2807	(45.8%)	2218	(46.4%)	
Yes, n	5885	(53.9%)	3324	(54.2%)	2561	(53.6%)	
Physical activity level‡	1.45	(1.4–1.51)	1.45	(1.4–1.51)	1.45	(1.4–1.51)	*0.5738*
Total energy intake (kcal/day) †	1735	(1504–1980)	1735	(1501–1982)	1735	(1509–1979)	*0.6589*
Energy intake excluding energy from alcoholic beverages (kcal/day) †	1673	(1437–1921)	1651	(1413–1905)	1701	(1470–1942)	*<0.0001*
Carbohydrate intake (g/day) †	244	(204.8–284.9)	240.6	(201–282)	248.3	(209.9–288.1)	*<0.0001*
Protein intake (g/day) †	54.13	(45.8–63.5)	53.81	(45.44–63.31)	54.57	(46.19–63.82)	*0.0026*
Fat intake (g/day) †	43.39	(35.11–53.32)	43.2	(34.89–53.22)	43.66	(35.49–53.55)	*0.0257*

Medians and interquartile ranges are shown for continuous variables. BW, body weight. †Standardized for 60 kg of BW. ‡ Ratio of total energy expenditure to basal metabolic rate. *p*-values determined using chi-square or Mann–Whitney U test. Data missing for years of education (n = 30), current smoking (n = 1), diabetes mellitus (n = 2), hyperlipidemia (n = 5), hypertension (n = 1), ischemic disease (n = 4), stroke (n = 1), and physical activity level (n = 160).

### 3.2. Interactive effects between the *ALDH2* rs671 polymorphism and ethanol consumption on energy and macronutrient intake

**Fig. 1:**
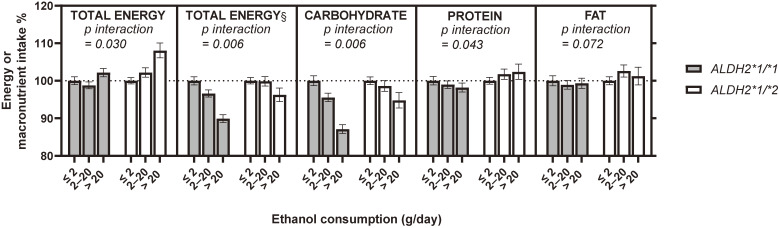
Interaction between *ALDH2* rs671 polymorphism and ethanol consumption on energy and macronutrient intakes. The bars represent ratios of least-square geometric means computed with general linear regression models using the separated database for each genotype, adjusted for age, sex, years of education, ethanol consumption (continuous variable), smoking status, medical history, dietary restrictions, and physical activity levels. Error bars represent 95% confidence intervals. § Energy intake excluding energy from alcoholic beverages. *p interaction* indicates the probability of the interactive effect tested using interaction terms between the *ALDH2* rs671 polymorphism and ethanol consumption (continuous variable) in general linear regression models including covariates of sex, years of education, ethanol consumption (continuous variable), smoking status, medical history, dietary restrictions, physical activity levels, and *ALDH2* polymorphism (see Model B in Table S4).

In line with our hypothesis, multivariate regression analyses revealed significant interactive effects between rs671 and ethanol consumption on energy and macronutrient intake (*p* for interaction = 0.005–0.072) (Table S4), although the effects on protein and fat intake were marginal (*p* for interaction = 0.043–0.072) (Table S4). To visualize the interactive effects, the least square geometric means were computed and are presented in Fig. 1. Sensitivity analyses with an additional covariate, body height (Table S5 and Fig. S1) or body fat percentage (Table S6 and Fig. S2), revealed similar results. Further analysis with stratification by sex largely attenuated the statistical significance of the interaction term; however, similar tendencies were found for both sexes (Tables S7 and S8, Figs. S3 and S4).

**Fig. 2:**
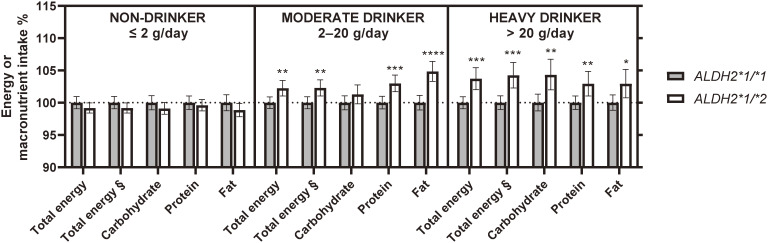
Difference in energy and macronutrient intake between *ALDH2* rs671 genotypes by levels of ethanol consumption. Differences in energy and macronutrient intakes between the *ALDH2* rs671 genotypes were tested in stratified cohorts by ethanol consumption levels. The general linear regression models included age, sex, ethanol consumption amount, education years, current smoking status, medical history, dietary restrictions, and physical activity level as covariates (see Model III in Table S9). Error bars represent 95% confidence intervals. § Energy intake excluding energy from alcoholic beverages. * *p* < *0.05*, ** *p* < *0.01*, *** *p* < *0.001*, and **** *p* < *0.0001* between ALDH2*1/*1 and ALDH2*1/*2.

To visualize the genotypic effect, an intergenic comparisons of energy and macronutrient intakes within the cohort stratified according to ethanol consumption level, are shown in Fig. 2 and S5 (by sex). ALDH2*1/*2 carriers among moderate and heavy drinkers had higher energy intake than ALDH2*1/*1 carriers, whereas there was no such association among non-drinkers (Table S9 and Fig. 2). The difference in total energy intake between the genotypes was estimated to be >60 kcal/day in heavy drinkers in models including ethanol consumption as a covariate (models II and III, Table S9). Similarly, energy intake excluding energy from alcoholic beverages showed a difference of 70–80 kcal/day. The estimated difference in carbohydrate intake was >10 g/day in heavy drinkers. Protein and fat intake were estimated to be between 1–2 g/day in moderate and heavy drinkers.

**Fig. 3:**
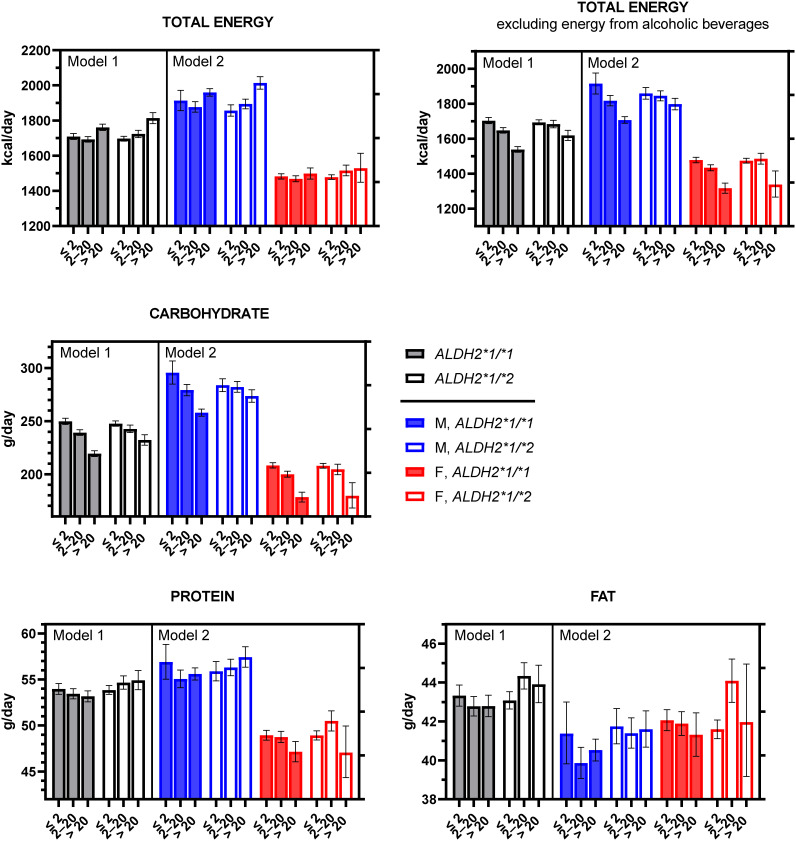
Estimated energy and macronutrient intakes based on ALDH2 rs671 genotypes and ethanol consumption levels. The bars represent least-square geometric means (LSGMs) computed with general linear regression models; incorporated within model 1 was an interaction term of [rs671*ethanol consumption categories (<2, 2–20, and >20 g/day, as a categorical variable)] with covariates of age, sex, years of education, smoking status, medical history, dietary restrictions, and physical activity levels. Meanwhile, model 2 included [rs671ethanol consumption categories (<2, 2–20, and >20 g/day, as a categorical variable)*sex] term, age, sex, years of education, smoking status, medical history, dietary restrictions, and physical activity levels. Error bars represent 95% confidence intervals. The LSGMs model 2 are exhibited in the form of kcal or g/day/65.7 kg body weight for males (M) or kcal or g/day/52.5 kg body weight for females (F).

Finally, the estimated geometric mean derived from the entire database is presented in Fig. 3, utilizing the actual units of the outcomes (kcal/day or g/day).

## 4. Discussion

As hypothesized, energy and macronutrient intakes in individuals with habitual drinking habits were higher in ALDH2*1/*2 than in ALDH2*1/*1 carriers. The associations were consistent for all components, that is, energy intake excluding energy from alcoholic beverages, and carbohydrate, protein, and fat intakes. The associations were maintained after statistical adjustment for ethanol consumption and in stratified analyses by alcohol consumption level. To the best of our knowledge, this is the first study to report that individuals with low ALDH2 activity may require extra energy and macronutrient intakes when they consume alcoholic beverages. This may explain the trend that habitual drinking of alcoholic beverages among ALDH2*2 carriers is less likely to increase serum triglycerides and visceral fat. Recent studies have reported higher sweet taste, confection, and carbohydrate preferences in ALDH2*2 carriers than in non-carriers (ALDH2*1/*1) [51–53]. However, these findings seem irrelevant to the phenomenon observed in the current study because the associations were largely attenuated after adjustment for drinking behavior. These previous studies probably detected differences in food preferences resulting from the drinking habit, as rs671 strongly reduces the preference for alcoholic beverages.

Detoxification by GSH [54] represents a possible alternative acetaldehyde metabolic pathway in individuals with impaired ALDH2 activity. In 2009, Endo et al. [40] reported that transgenic mice with impaired ALDH activity due to overexpression of inactive ALDH2 demonstrated elevated blood levels of aldehydes and suppressed weight gain and adipose tissue mass, but unexpectedly maintained cardiac function and showed surprisingly high tolerance to oxidative stress from ischemia and reperfusion. According to their transcriptome and metabolome analyses, this hormesis-like phenomenon was due to a strong induction of GSH biosynthesis triggered by the phosphorylation of eukaryotic initiation factor 2 (eIF2), a regulatory node that controls the initiation of protein synthesis. Phosphorylated eIF2 activates activating transcription factor 4, which increases the expression of gene cluster encoding enzymes involved in amino acid biosynthesis and transport, ultimately providing precursor amino acids for GSH biosynthesis. Furthermore, although the mechanism remains to be fully unraveled, an increase in the supply of NADPH resulting from activation of the pentose phosphate pathway, which was evidenced in this study, seemed to enhance the GSH-mediated detoxification system [40]. Thus, the findings of this study suggest that individuals with suppressed ALDH activity require nutrients to compensate for the increased detoxification capacity when exposed to aldehydes. The finding by Guillot et al. that heat production and the respiratory quotient were suppressed in *Aldh2*^−/−^ mice after consumption of 5 g/kg ethanol [55] is consistent with the above hypothesis; heat production should decrease if energy sources are geared toward GSH production. Moreover, the pentose phosphate pathway is characterized by not producing ATP, but CO_2_, which lowers the respiratory quotient. However, sympathetic stimulation by acetaldehyde may influence with the energy balance as epinephrine enhances energy consumption [56]. Increased sympathetic nervous system activity has been reported in ALDH2*1/*2 carriers after alcohol consumption [57–60], in whom catecholamines possibly contribute to blood pressure homeostasis, i.e., minimizing the decrease in blood pressure due to the vasodilatory effect of acetaldehyde [61].

The current study validated for the first time the hypothesis that individuals with inhibited ALDH activity require more energy when they consume ethanol, possibly to activate the GSH detoxification system, an alternative detoxification pathway of ALDH. However, several limitations should be addressed. First, since this is an observational and cross-sectional study, our hypothesis should be confirmed by other methods, including intervention studies. Second, the accuracy in the amount of alcohol consumption and energy and macronutrient intake was limited because the calculations were performed using self-administered questionnaires. Bongers et al. pointed out discrepancies between self-reported and actual alcohol consumption levels; alcohol consumption in heavy drinkers tends to be overreported in men and underreported in women, particularly in older women with lower educational attainment [62]. Third, because of the small number of heavy drinkers among ALDH2*1/*2 carriers, stronger associations may have been masked. Furthermore, a larger population sample is required to examine ALDH2*2/*2 carriers, who were excluded from this study. Fourth, the rs1229984 polymorphism of *ADH1B* gene, which strongly affects the production rate of acetaldehyde from ethanol, is not in the database; therefore, we could not adjust for the impact. Fifth, since we used data from approximately 20% of the total subjects recruited in the J-MICC study, participant bias may have affected the study outcomes. For example, compared with the parent population, the present cohort included higher proportions of women and the elderly [42]. Lastly, this study included only men and women aged between 40–69 years living in Saga City; therefore, the generalizability of the study results is limited.

In conclusion, we performed an epidemiological analysis to examine a hypothesis based on previous animal research, and the present results suggested that increased energy and macronutrient intake are required in *ALDH2* rs671 variant allele, ALDH2*2, carriers who routinely consume alcoholic beverages. Although the mechanism needs to be further verified, this finding supports the hypothesis that individuals with low ALDH activity require induced GSH detoxification upon aldehyde overload. This study provides a possible scientific basis for understanding the mechanism of homeostasis associated with aldehyde loading, and suggests a novel phenotype of a common genetic polymorphism in East Asia.

## Supplementary information

**Additional file 1:** **Table S1.** Characteristics of non-drinkers (≤2 g/day) based on *ALDH2* rs671 polymorphism. **Table S2.** Characteristics of moderate drinkers (2–20 g/day) based on *ALDH2* rs671 polymorphism. **Table S3.** Characteristics of heavy drinkers (>20 g/day) based on *ALDH2* rs671 polymorphism. **Table S4.** Effects of *ALDH2* rs671 polymorphism, ethanol consumption, and interaction terms between energy and macronutrient intakes. **Table S5.** Effects of *ALDH2* rs671 polymorphism, ethanol consumption, and interaction terms between energy and macronutrient intakes (sensitivity analysis using a model including height as a covariate). **Figure S1.** Ratios of energy and macronutrient intakes estimated using a model with height as a covariate for sensitivity analysis. **Table S6.** Effects of ALDH2 rs671 polymorphism, ethanol consumption, and interaction terms between energy and macronutrient intakes (sensitivity analysis using a model including body fat percentage as a covariate). **Figure S2.** Ratios of energy and macronutrient intakes estimated using a model with body fat percentage as a covariate in a sensitivity analysis. **Table S7.** Effects of *ALDH2* rs671 polymorphism, ethanol consumption, and interaction terms between energy and macronutrient intakes in male participants. **Figure S3.** Ratios of energy and macronutrient intakes estimated in male participants, as sensitivity analysis. **Table S8.** Effects of *ALDH2* rs671 polymorphism, ethanol consumption, and interaction terms between energy and macronutrient intakes in female participants. **Figure S4.** Ratios of energy and macronutrient intakes estimated in female participants, as sensitivity analysis. **Figure S5.** Sensitivity analysis. **Table S9.** Estimated energy and macronutrient intakes based on *ALDH2* rs671 polymorphism and ethanol consumption levels.

## Declaration

### Availability of data and materials

The datasets generated and/or analyzed in the current study are not publicly available to protect the privacy of participants, but they are available from the corresponding author upon reasonable request.
