# Supplementary material for: ALDH2 rs671 variant allele is associated with higher energy intake in middle-aged and elderly Japanese who routinely consume alcohol
Source: Environ Health Prev Med. 2023 May 11;28:29. doi: 10.1265/ehpm.22-00276 (PMC10188283; doi:10.1265/ehpm.22-00276)
Supplement: Supplementary file 1 — Additional file 1: Table S1. Characteristics of non-drinkers (≤2 g/day) based on ALDH2 rs671 polymorphism. Table S2. Characteristics of moderate drinkers (2–20 g/day) based on ALDH2 rs671 polymorphism. Table S3. Characteristics of heavy drinkers (>20 g/day) based on ALDH2 rs671 polymorphism. Table S4. Effects of ALDH2 rs671 polymorphism, ethanol consumption, and interaction terms between energy and macronutrient intakes. Table S5. Effects of ALDH2 rs671 polymorphism, ethanol consumption, and interaction terms between energy and macronutrient intakes (sensitivity analysis using a model including height as a covariate). Figure S1. Ratios of energy and macronutrient intakes estimated using a model with height as a covariate for sensitivity analysis. Table S6. Effects of ALDH2 rs671 polymorphism, ethanol consumption, and interaction terms between energy and macronutrient intakes (sensitivity analysis using a model including body fat percentage as a covariate). Figure S2. Ratios of energy and macronutrient intakes estimated using a model with body fat percentage as a covariate in a sensitivity analysis. Table S7. Effects of ALDH2 rs671 polymorphism, ethanol consumption, and interaction terms between energy and macronutrient intakes in male participants. Figure S3. Ratios of energy and macronutrient intakes estimated in male participants, as sensitivity analysis. Table S8. Effects of ALDH2 rs671 polymorphism, ethanol consumption, and interaction terms between energy and macronutrient intakes in female participants. Figure S4. Ratios of energy and macronutrient intakes estimated in female participants, as sensitivity analysis. Figure S5. Sensitivity analysis. Table S9. Estimated energy and macronutrient intakes based on ALDH2 rs671 polymorphism and ethanol consumption levels. [file ehpm-28-029-s001.docx]

| **Table S1. Characteristics of non-drinkers (≤ 2 g/day) based on *ALDH2* rs671 polymorphism** | | | | | | | | |
| --- | --- | --- | --- | --- | --- | --- | --- | --- |
|  | | Total | | *ALDH2*1/*1* | | *ALDH2*1/*2* | | *p-*value |
| All, n | | 4895 | (100%) | 2030 | (41.5%) | 2865 | (58.5%) |  |
| Age, years (IQR) | | 58 | (50–63) | 58 | (51–64) | 57 | (50–63) | 0.082 |
| Sex | |  |  |  |  |  |  | < 0.0001 |
|  | Men, n | 830 | (17%) | 204 | (10.1%) | 626 | (21.9%) |  |
|  | Women, n | 4065 | (83%) | 1826 | (90%) | 2239 | (78.2%) |  |
| Years of education | |  |  |  |  |  |  | 0.4226 |
|  | ≤ 9 years, n | 297 | (6.1%) | 116 | (5.7%) | 181 | (6.3%) |  |
|  | 9–12 years, n | 2666 | (54.7%) | 1125 | (55.7%) | 1541 | (53.9%) |  |
|  | > 12 years, n | 1915 | (39.3%) | 780 | (38.6%) | 1135 | (39.7%) |  |
| Ethanol consumption (g/day) † | | 0.0 | (0–0) | 0.0 | (0–0) | 0.0 | (0–0) | < 0.0001 |
| Current smoking | |  |  |  |  |  |  | < 0.0001 |
|  | Yes, n | 542 | (11.1%) | 159 | (7.8%) | 383 | (13.4%) |  |
|  | No, n | 4352 | (88.9%) | 1871 | (92.2%) | 2481 | (86.6%) |  |
| BW (kg) (IQR) | | 54.0 | (48.8–60.9) | 53.8 | (48.8–60) | 54.3 | (48.9–61.6) | 0.0056 |
| Medical history | |  |  |  |  |  |  |  |
| Liver cirrhosis | |  |  |  |  |  |  | 0.0382 |
|  | No, n | 4867 | (99.4%) | 2013 | (99.2%) | 2854 | (99.6%) |  |
|  | Yes, n | 28 | (0.6%) | 17 | (0.8%) | 11 | (0.4%) |  |
| Diabetes mellitus | |  |  |  |  |  |  | 0.2755 |
|  | No, n | 4596 | (93.9%) | 1897 | (93.5%) | 2699 | (94.2%) |  |
|  | Yes, n | 299 | (6.1%) | 133 | (6.6%) | 166 | (5.8%) |  |
| Hyperlipidaemia | |  |  |  |  |  |  | 0.5013 |
|  | No, n | 3887 | (79.5%) | 1619 | (80%) | 2268 | (79.2%) |  |
|  | Yes, n | 1003 | (20.5%) | 406 | (20.1%) | 597 | (20.8%) |  |
| Hypertension | |  |  |  |  |  |  | 0.0011 |
|  | No, n | 4034 | (82.4%) | 1630 | (80.3%) | 2404 | (83.9%) |  |
|  | Yes, n | 861 | (17.6%) | 400 | (19.7%) | 461 | (16.1%) |  |
| Ischemic heart disease | |  |  |  |  |  |  | 0.3830 |
|  | No, n | 4748 | (97%) | 1973 | (97.3%) | 2775 | (96.9%) |  |
|  | Yes, n | 145 | (3%) | 55 | (2.7%) | 90 | (3.1%) |  |
| Stroke | |  |  |  |  |  |  | 0.1232 |
|  | No, n | 4817 | (98.4%) | 1991 | (98.1%) | 2826 | (98.6%) |  |
|  | Yes, n | 78 | (1.6%) | 39 | (1.9%) | 39 | (1.4%) |  |
| Dietary restrictions | |  |  |  |  |  |  |  |
| Salt | |  |  |  |  |  |  | < 0.0001 |
|  | No, n | 1907 | (39%) | 717 | (35.3%) | 1190 | (41.5%) |  |
|  | Yes, n | 2988 | (61%) | 1313 | (64.7%) | 1675 | (58.5%) |  |
| Calories | |  |  |  |  |  |  | 0.1359 |
|  | No, n | 2495 | (51%) | 1009 | (49.7%) | 1486 | (51.9%) |  |
|  | Yes, n | 2400 | (49%) | 1021 | (50.3%) | 1379 | (48.1%) |  |
| Sugar | |  |  |  |  |  |  | 0.0153 |
|  | No, n | 2786 | (56.9%) | 1114 | (54.9%) | 1672 | (58.4%) |  |
|  | Yes, n | 2109 | (43.1%) | 916 | (45.1%) | 1193 | (41.6%) |  |
| Fat | |  |  |  |  |  |  | 0.0131 |
|  | No, n | 1965 | (40.1%) | 773 | (38.1%) | 1192 | (41.6%) |  |
|  | Yes, n | 2930 | (59.9%) | 1257 | (61.9%) | 1673 | (58.4%) |  |
| Physical activity level‡ (IQR) | | 1.45 | (1.4–1.51) | 1.45 | (1.41–1.51) | 1.45 | (1.4–1.51) | 0.2364 |
| Total energy intake (kcal/day) † (IQR) | | 1724 | (1492–1964) | 1737 | (1492–1978) | 1716 | (1492–1953) | 0.2144 |
| Energy intake excluding energy from alcoholic beverages (kcal/day) † (IQR) | | 1723 | (1492–1964) | 1736 | (1489–1977) | 1716 | (1492–1952) | 0.2385 |
| Carbohydrate intake (g/day) † (IQR) | | 248.2 | (210.0–285.8) | 248.3 | (210.0–286.8) | 248.0 | (210.0–285.4) | 0.9789 |
| Protein intake (g/day) † (IQR) | | 55.8 | (47.42–65.63) | 56.6 | (47.86–66.04) | 55.2 | (47.15–65.35) | 0.0201 |
| Fat intake (g/day) † (IQR) | | 46.3 | (37.75–56.25) | 47.3 | (38.35–57.41) | 45.7 | (37.44–55.38) | < 0.0001 |
| IQR, interquartile range. BW, body weight. †Standardized for 60 kg of BW. ‡ Ratio of total energy expenditure to basal metabolic rate. *p*-values determined using chi-square or Mann–Whitney U test.  Non-drinkers were defined as participants with ethanol consumption ≤ 2 g/day. Data missing for years of education (n = 17), current smoking (n = 1), hyperlipidaemia (n = 5), ischemic disease (n = 2), and physical activity level (n = 82). | | | | | | | | |

| **Table S2. Characteristics of moderate drinkers (2–20 g/day) based on *ALDH2* rs671 polymorphism** | | | | | | | | |
| --- | --- | --- | --- | --- | --- | --- | --- | --- |
|  | | Total | | *ALDH2*1/*1* | | *ALDH2*1/*2* | | *p-*value |
| All, n | | 3352 | (100%) | 2085 | (62.2%) | 1267 | (37.8%) |  |
| Age, years (IQR) | | 56 | (48–62) | 55 | (48–62) | 56.0 | (48–62) | 0.0820 |
| Sex | |  |  |  |  |  |  | < 0.0001 |
|  | Men, n | 1532 | (45.7%) | 717 | (34.4%) | 815 | (64.3%) |  |
|  | Women, n | 1820 | (54.3%) | 1368 | (65.6%) | 452 | (35.7%) |  |
| Years of education | |  |  |  |  |  |  | 0.0962 |
|  | ≤ 9 years, n | 155 | (4.6%) | 93 | (4.5%) | 62 | (4.9%) |  |
|  | 9–12 years, n | 1539 | (46%) | 987 | (47.5%) | 552 | (43.6%) |  |
|  | > 12 years, n | 1650 | (49.3%) | 999 | (48.1%) | 651 | (51.5%) |  |
| Ethanol consumption (g/day) † | | 7.8 | (4.3–12.9) | 7.8 | (4.3–13.3) | 7.7 | (4.4–12.3) | < 0.0001 |
| Current smoking | |  |  |  |  |  |  | < 0.0001 |
|  | Yes, n | 581 | (17.3%) | 300 | (14.4%) | 281 | (22.2%) |  |
|  | No, n | 2771 | (82.7%) | 1785 | (85.6%) | 986 | (77.8%) |  |
| BW (kg) (IQR) | | 58.3 | (51.4–66.6) | 56.3 | (50.6–64.5) | 61.5 | (53.3–68.9) | 0.0056 |
| Medical history | |  |  |  |  |  |  |  |
| Liver cirrhosis | |  |  |  |  |  |  | > 0.9999 |
|  | No, n | 3349 | (99.9%) | 2083 | (99.9%) | 1266 | (99.9%) |  |
|  | Yes, n | 3 | (0.1%) | 2 | (0.1%) | 1 | (0.1%) |  |
| Diabetes mellitus | |  |  |  |  |  |  | 0.2463 |
|  | No, n | 3183 | (95%) | 1987 | (95.3%) | 1196 | (94.4%) |  |
|  | Yes, n | 169 | (5%) | 98 | (4.7%) | 71 | (5.6%) |  |
| Hyperlipidaemia | |  |  |  |  |  |  | 0.9565 |
|  | No, n | 2734 | (81.6%) | 1700 | (81.5%) | 1034 | (81.6%) |  |
|  | Yes, n | 618 | (18.4%) | 385 | (18.5%) | 233 | (18.4%) |  |
| Hypertension | |  |  |  |  |  |  | 0.5153 |
|  | No, n | 2764 | (82.5%) | 1712 | (82.2%) | 1052 | (83%) |  |
|  | Yes, n | 587 | (17.5%) | 372 | (17.9%) | 215 | (17%) |  |
| Ischemic heart disease | |  |  |  |  |  |  | 0.2555 |
|  | No, n | 3259 | (97.3%) | 2032 | (97.5%) | 1227 | (96.8%) |  |
|  | Yes, n | 92 | (2.8%) | 52 | (2.5%) | 40 | (3.2%) |  |
| Stroke | |  |  |  |  |  |  | 0.1242 |
|  | No, n | 3300 | (98.5%) | 2047 | (98.2%) | 1253 | (98.9%) |  |
|  | Yes, n | 51 | (1.5%) | 37 | (1.8%) | 14 | (1.1%) |  |
| Dietary restrictions | |  |  |  |  |  |  |  |
| Salt | |  |  |  |  |  |  | 0.0167 |
|  | No, n | 1499 | (44.7%) | 899 | (43.1%) | 600 | (47.4%) |  |
|  | Yes, n | 1853 | (55.3%) | 1186 | (56.9%) | 667 | (52.6%) |  |
| Calories | |  |  |  |  |  |  | 0.6086 |
|  | No, n | 1833 | (54.7%) | 1133 | (54.3%) | 700 | (55.3%) |  |
|  | Yes, n | 1519 | (45.3%) | 952 | (45.7%) | 567 | (44.8%) |  |
| Sugar | |  |  |  |  |  |  | 0.8748 |
|  | No, n | 2042 | (60.9%) | 1268 | (60.8%) | 774 | (61.1%) |  |
|  | Yes, n | 1310 | (39.1%) | 817 | (39.2%) | 493 | (38.9%) |  |
| Fat | |  |  |  |  |  |  | 0.0099 |
|  | No, n | 1558 | (46.5%) | 933 | (44.8%) | 625 | (49.3%) |  |
|  | Yes, n | 1794 | (53.5%) | 1152 | (55.3%) | 642 | (50.7%) |  |
| Physical activity level‡ (IQR) | | 1.45 | (1.41–1.51) | 1.45 | (1.41–1.51) | 1.45 | (1.41–1.51) | 0.2364 |
| Total energy intake (kcal/day) † (IQR) | | 1721 | (1498–1955) | 1702 | (1493–1934) | 1745 | (1509–1984) | 0.2144 |
| Energy intake excluding energy from alcoholic beverages (kcal/day) † (IQR) | | 1676 | (1456–1917) | 1662 | (1450–1897) | 1705 | (1471–1943) | 0.0026 |
| Carbohydrate intake (g/day) † (IQR) | | 244.3 | (207.1–285.1) | 240.6 | (205.1–279.7) | 251.7 | (211.1–292.1) | 0.9789 |
| Protein intake (g/day) † (IQR) | | 54.19 | (46.05–62.66) | 54.25 | (46.4–62.62) | 53.98 | (45.59–62.78) | 0.0201 |
| Fat intake (g/day) † (IQR) | | 43.38 | (35.34–52.97) | 44.05 | (36.17–53.35) | 42.44 | (33.88–51.97) | < 0.0001 |
| IQR, interquartile range. BW, body weight. †Standardized for 60 kg of BW. *p*-values determined using chi-square or Mann–Whitney U test. Fisher’s exact test was employed when the expected value of 25% of cells was <5. ‡ Ratio of total energy expenditure to basal metabolic rate.  Moderate drinkers were defined as participants with ethanol consumption of 2–20 g g/day. Data missing for years of education (n = 8), hypertension (n = 1), ischemic disease (n = 1), stroke (n = 1), and physical activity level (n = 36). | | | | | | | | |

| **Table S3. Characteristics of heavy drinkers (> 20 g/day) based on *ALDH2* rs671 polymorphism** | | | | | | | | |
| --- | --- | --- | --- | --- | --- | --- | --- | --- |
|  |  | Total | | *ALDH2*1/*1* | | *ALDH2*1/*2* | | *p-*value |
| All, n | | 2663 | (100%) | 2016 | (75.7%) | 647 | (24.3%) |  |
| Age, years (IQR) | | 57 | (49–63) | 57 | (50–63) | 56 | (49–63) | 0.2737 |
| Sex | |  |  |  |  |  |  | < 0.0001 |
|  | Men, n | 2210 | (83%) | 1624 | (80.6%) | 586 | (90.6%) |  |
|  | Women, n | 453 | (17%) | 392 | (19.4%) | 61 | (9.4%) |  |
| Years of education | |  |  |  |  |  |  | 0.0606 |
|  | ≤ 9 years, n | 193 | (7.3%) | 146 | (7.3%) | 47 | (7.3%) |  |
|  | 9–12 years, n | 1217 | (45.8%) | 946 | (47%) | 271 | (41.9%) |  |
|  | > 12 years, n | 1248 | (47%) | 919 | (45.7%) | 329 | (50.9%) |  |
| Ethanol consumption (g/day) † | | 39.4 | (28.3–56.8) | 40.4 | (29.1–58.7) | 36.3 | (25.8–49.9) | < 0.0001 |
| Current smoking | |  |  |  |  |  |  | 0.0011 |
|  | Yes, n | 1056 | (39.7%) | 764 | (37.9%) | 292 | (45.1%) |  |
|  | No, n | 1607 | (60.4%) | 1252 | (62.1%) | 355 | (54.9%) |  |
| BW (kg) (IQR) | | 63.5 | (56.2–70.5) | 63.5 | (55.8–70.6) | 63.5 | (57.3–69.8) | 0.5159 |
| Medical history | |  |  |  |  |  |  |  |
| Liver cirrhosis | |  |  |  |  |  |  | 0.7345 |
|  | No, n | 2652 | (99.6%) | 2008 | (99.6%) | 644 | (99.5%) |  |
|  | Yes, n | 11 | (0.4%) | 8 | (0.4%) | 3 | (0.5%) |  |
| Diabetes mellitus | |  |  |  |  |  |  | 0.0416 |
|  | No, n | 2423 | (91.1%) | 1821 | (90.4%) | 602 | (93%) |  |
|  | Yes, n | 238 | (8.9%) | 193 | (9.6%) | 45 | (7%) |  |
| Hyperlipidaemia | |  |  |  |  |  |  | 0.0294 |
|  | No, n | 2179 | (81.8%) | 1631 | (80.9%) | 548 | (84.7%) |  |
|  | Yes, n | 484 | (18.2%) | 385 | (19.1%) | 99 | (15.3%) |  |
| Hypertension | |  |  |  |  |  |  | 0.1440 |
|  | No, n | 1975 | (74.2%) | 1481 | (73.5%) | 494 | (76.4%) |  |
|  | Yes, n | 688 | (25.8%) | 535 | (26.5%) | 153 | (23.7%) |  |
| Ischemic heart disease | |  |  |  |  |  |  | 0.4582 |
|  | No, n | 2576 | (96.8%) | 1947 | (96.6%) | 629 | (97.2%) |  |
|  | Yes, n | 86 | (3.2%) | 68 | (3.4%) | 18 | (2.8%) |  |
| Stroke | |  |  |  |  |  |  | 0.6039 |
|  | No, n | 2620 | (98.4%) | 1982 | (98.3%) | 638 | (98.6%) |  |
|  | Yes, n | 43 | (1.6%) | 34 | (1.7%) | 9 | (1.4%) |  |
| Dietary restrictions | |  |  |  |  |  |  |  |
| Salt | |  |  |  |  |  |  | 0.0005 |
|  | No, n | 1406 | (52.8%) | 1026 | (50.9%) | 380 | (58.7%) |  |
|  | Yes, n | 1257 | (47.2%) | 990 | (49.1%) | 267 | (41.3%) |  |
| Calories | |  |  |  |  |  |  | 0.1445 |
|  | No, n | 1631 | (61.3%) | 1219 | (60.5%) | 412 | (63.7%) |  |
|  | Yes, n | 1032 | (38.8%) | 797 | (39.5%) | 235 | (36.3%) |  |
| Sugar | |  |  |  |  |  |  | 0.0375 |
|  | No, n | 1649 | (61.9%) | 1226 | (60.8%) | 423 | (65.4%) |  |
|  | Yes, n | 1014 | (38.1%) | 790 | (39.2%) | 224 | (34.6%) |  |
| Fat | |  |  |  |  |  |  | 0.0010 |
|  | No, n | 1502 | (56.4%) | 1101 | (54.6%) | 401 | (62%) |  |
|  | Yes, n | 1161 | (43.6%) | 915 | (45.4%) | 246 | (38%) |  |
| Physical activity level‡ (IQR) | | 1.45 | (1.4–1.51) | 1.45 | (1.4–1.51) | 1.44 | (1.4–1.5) | 0.9768 |
| Total energy intake (kcal/day) † (IQR) | | 1775 | (1537–2058) | 1767 | (1524–2040) | 1804 | (1594–2106) | 0.0019 |
| Energy intake excluding energy from alcoholic beverages (kcal/day) † (IQR) | | 1563 | (1325–1836) | 1543 | (1301–1814) | 1623 | (1396–1884) | < 0.0001 |
| Carbohydrate intake (g/day) † (IQR) | | 235.5 | (190.7–282.0) | 232.0 | (186.9–278.8) | 245.5 | (205.6–293.9) | < 0.0001 |
| Protein intake (g/day) † (IQR) | | 51.11 | (43.28–60.23) | 50.66 | (43.03–59.84) | 52.66 | (44.23–60.76) | 0.0238 |
| Fat intake (g/day) † (IQR) | | 37.82 | (31.59–46.65) | 37.81 | (31.38–46.62) | 37.85 | (32.03–46.71) | 0.7075 |
| IQR, interquartile range. BW, body weight. †Standardized for 60 kg of BW. ‡ Ratio of total energy expenditure to basal metabolic rate. *p*-values determined using chi-square or Mann–Whitney U test. Fisher’s exact test was employed when the expected value of 25% of cells was < 5.  Heavy drinkers were defined as ethanol consumption > 20 g/day. Data missing for years of education (n = 5), diabetes mellitus (n = 2), ischemic disease (n = 1), and physical activity level (n = 42). | | | | | | | | |

| **Table S4. Effects of *ALDH2* rs671 polymorphism, ethanol consumption, and interaction terms between energy and macronutrient intakes** | | | | | | | | |
| --- | --- | --- | --- | --- | --- | --- | --- | --- |
| Outcome variables  (log-transformed) | | *ALDH2* rs671 polymorphism  (reference=*ALDH2*1/*1*) | | Ethanol consumption  (g/day) | | Interaction terms between  *ALDH2* and ethanol consumption | |  |
|  |  | β | *p-*value | β | *p-*value | β | *p-*value |  |
| Total energy intake (kcal/day) | Model A | 0.01003 | 0.0396 | −0.00167 | < 0.0001 | 0.00048 | 0.0244 |  |
|  | Model B | 0.00970 | 0.0444 | −0.00177 | < 0.0001 | 0.00046 | 0.0296 |  |
| Energy intake excluding energy from alcoholic beverages (kcal/day) | Model A | 0.01029 | 0.0436 | −0.00166 | < 0.0001 | 0.00062 | 0.0052 |  |
|  | Model B | 0.01029 | 0.0517 | −0.00155 | < 0.0001 | 0.00063 | 0.0058 |  |
| Carbohydrate intake (g/day) | Model A | 0.00698 | 0.2444 | 0.00145 | < 0.0001 | 0.00073 | 0.0053 |  |
|  | Model B | 0.00736 | 0.2167 | 0.00132 | < 0.0001 | 0.00072 | 0.0061 |  |
| Protein intake (g/day) | Model A | 0.00870 | 0.106 | −0.00026 | 0.2163 | 0.00046 | 0.0526 |  |
|  | Model B | 0.00786 | 0.1409 | −0.00038 | 0.0769 | 0.00048 | 0.0429 |  |
| Fat intake (g/day) | Model A | 0.01175 | 0.058 | −0.00050 | 0.0435 | 0.00050 | 0.0652 |  |
|  | Model B | 0.00957 | 0.1221 | −0.00053 | 0.0315 | 0.00049 | 0.0717 |  |
| β, partial regression coefficient.  Model A included *ALDH2* rs671 polymorphism, ethanol consumption, and interaction terms between *ALDH2* and ethanol consumption as explanatory variables, with sex and age as covariates. Model B included explanatory variables and covariates in model A and additional covariates of years of education, current smoking status, dietary restrictions, medical history, and physical activity level. Energy and macronutrient intakes (outcomes) and ethanol consumption are standardized for 60 kg of BW. | | | | | | | | |

| **Table S5. Effects of *ALDH2* rs671 polymorphism, ethanol consumption, and interaction terms between** **energy and macronutrient intakes (sensitivity analysis using a model including height as a covariate)** | | | | | | | |
| --- | --- | --- | --- | --- | --- | --- | --- |
| Outcome variables  (log-transformed) | *ALDH2* rs671 polymorphism  (reference=*ALDH2*1/*1*) | | Ethanol consumption  (g/day) | | Interaction term between  *ALDH2* and ethanol consumption | |  |
|  | β | *p-*value | β | *p-*value | β | *p-*value |  |
| Total energy intake (kcal/day) | 0.00746 | 0.1107 | −0.00177 | < 0.0001 | 0.00053 | 0.0104 |  |
| Energy intake excluding energy from alcoholic beverages (kcal/day) | 0.00747 | 0.1268 | −0.00093 | < 0.0001 | 0.00068 | 0.0015 |  |
| Carbohydrate intake (g/day) | 0.00495 | 0.3947 | 0.00132 | < 0.0001 | 0.00079 | 0.0020 |  |
| Protein intake (g/day) | 0.00560 | 0.2821 | −0.00038 | 0.0683 | 0.00054 | 0.0179 |  |
| Fat intake (g/day) | 0.00741 | 0.2235 | −0.00053 | 0.0282 | 0.00055 | 0.0385 |  |
| β, partial regression coefficient; BW, body weight.  General linear regression model included covariates of model B (Table S4) and body height.  Energy and macronutrient intakes (outcomes) and ethanol consumption are standardized for 60 kg of BW. | | | | | | | |

Figure S1. Ratios of energy and macronutrient intakes estimated using a model with height as a covariate for sensitivity analysis.

Ratios of the least square means of energy and macronutrient intakes, with 95% confidence interval, based on *ALDH2* rs671 polymorphism and ethanol consumption levels are shown. General linear regression model included age, sex, years of education, ethanol consumption (continuous variable), smoking status, medical history, dietary restrictions, physical activity level, and height as covariates. *P* for interaction was determined using interaction terms between *ALDH2* polymorphism and ethanol consumption (continuous variable) in the total study population. Macronutrient intakes (outcomes) and ethanol consumption are standardized for 60 kg of BW.

| **Table S6. Effects of ALDH2 rs671 polymorphism, ethanol consumption, and interaction terms between energy and macronutrient intakes (sensitivity analysis using a model including body fat percentage as a covariate)** | | | | | | | |
| --- | --- | --- | --- | --- | --- | --- | --- |
| Outcome variables  (log-transformed) | *ALDH2* rs671 polymorphism  (reference=*ALDH2*1/*1*) | | Ethanol consumption  (g/day) | | Interaction term between  *ALDH2* and ethanol consumption | |  |
|  | β | *p-*value | β | *p-*value | β | *p-*value |  |
| Total energy intake (kcal/day) | 0.00939 | 0.0514 | −0.00177 | < 0.0001 | 0.00047 | 0.0273 |  |
| Energy intake excluding energy from alcoholic beverages (kcal/day) | 0.00949 | 0.0596 | −0.000941 | < 0.0001 | 0.00062 | 0.0052 |  |
| Carbohydrate intake (g/day) | 0.00707 | 0.2351 | 0.00133 | < 0.0001 | 0.00072 | 0.0056 |  |
| Protein intake (g/day) | 0.00753 | 0.1579 | −0.00037 | 0.0807 | 0.00048 | 0.0399 |  |
| Fat intake (g/day) | 0.00924 | 0.1352 | −0.00053 | 0.0330 | 0.00050 | 0.0678 |  |
| β, partial regression coefficient; BW, body weight. Macronutrients (outcomes) and ethanol consumption are standardized for 60 kg of BW.  General linear regression model included covariates of model B (Table S4) and body fat percentage as covariates. | | | | | | | |

Figure S2. Ratios of energy and macronutrient intakes estimated using a model with body fat percentage as a covariate in a sensitivity analysis

Ratios of the least square means of energy and macronutrient intakes, with 95% confidence intervals, based on *ALDH2* rs671 polymorphism and ethanol consumption levels are shown. General linear regression model included age, sex, years of education, ethanol consumption (continuous variable), smoking status, medical history, dietary restrictions, physical activity level, and body fat percentage as covariates. *p* for interaction determined using interaction terms between *ALDH2* polymorphism and ethanol consumption (continuous variable) in the total study population. Macronutrients (outcomes) and ethanol consumption are standardized for 60 kg of BW.

Figure S3. Ratios of energy and macronutrient intakes estimated in male participants, as sensitivity analysis

Ratios of the least square means of energy and macronutrient intakes, with 95% confidence intervals, based on *ALDH2* rs671 polymorphism and ethanol consumption levels are shown. General linear regression model included age, years of education, ethanol consumption (continuous variable), smoking status, medical history, dietary restrictions, and physical activity level as covariates. *p* for interaction determined using interaction terms between *ALDH2* polymorphism and ethanol consumption (continuous variable) in the total study population. Macronutrients (outcomes) and ethanol consumption are standardized for 60 kg of BW.

| **Table S7. Effects of *ALDH2* rs671 polymorphism, ethanol consumption, and interaction terms between energy and macronutrient intakes in male participants** | | | | | | | |
| --- | --- | --- | --- | --- | --- | --- | --- |
| Outcome variables  (log-transformed) | *ALDH2* rs671 polymorphism  (reference=*ALDH2*1/*1*) | | Ethanol consumption  (g/day) | | Interaction term between  *ALDH2* and ethanol consumption | |  |
|  | β | *p-*value | β | *p-*value | β | *p-*value |  |
| Total energy intake (kcal/day) | 0.01395 | 0.1322 | 0.00184 | < 0.0001 | 0.00034 | 0.2195 |  |
| Energy intake excluding energy from alcoholic beverages (kcal/day) | 0.01502 | 0.1333 | −0.00086 | 0.0007 | 0.00045 | 0.1355 |  |
| Carbohydrate intake (g/day) | 0.00730 | 0.5367 | −0.00110 | 0.0002 | 0.00063 | 0.0758 |  |
| Protein intake (g/day) | 0.01348 | 0.1800 | 0.00059 | 0.0202 | 0.00040 | 0.1815 |  |
| Fat intake (g/day) | 0.03562 | 0.0026 | 0.00037 | 0.2163 | -0.00001 | 0.9810 |  |
| β, partial regression coefficient; BW, body weight.  The model included age, years of education, current smoking status, dietary restrictions, medical history, physical activity level, and body fat percentage as covariates. Macronutrients (outcomes) and ethanol consumption are standardized for 60 kg of BW. | | | | | | | |

Figure S4. Ratios of energy and macronutrient intakes estimated in female participants, as sensitivity analysis

Ratios of the least square means of energy and macronutrient intakes, with 95% confidence intervals, based on *ALDH2* rs671 polymorphism and ethanol consumption levels are shown. General linear regression model included age, years of education, ethanol consumption (continuous variable), smoking status, medical history, dietary restrictions, and physical activity level as covariates. *p* for interaction determined using interaction terms between *ALDH2* polymorphism and ethanol consumption (continuous variable) in the total study population. Macronutrients (outcomes) and ethanol consumption are standardized for 60 kg of BW.

| **Table S8. Effects of *ALDH2* rs671 polymorphism, ethanol consumption, and interaction terms between energy and macronutrient intakes in female participants** | | | | | | | |
| --- | --- | --- | --- | --- | --- | --- | --- |
| Outcome variables  (log-transformed) | *ALDH2* rs671 polymorphism  (reference=*ALDH2*1/*1*) | | Ethanol consumption  (g/day) | | Interaction term between  *ALDH2* and ethanol consumption | |  |
|  | β | *p-*value | β | *p-*value | β | *p-*value |  |
| Total energy intake (kcal/day) | 0.00668 | 0.2306 | 0.00132 | 0.0024 | 0.00058 | 0.2259 |  |
| Energy intake excluding energy from alcoholic beverages (kcal/day) | 0.00623 | 0.2722 | −0.00139 | 0.0016 | 0.00087 | 0.0755 |  |
| Carbohydrate intake (g/day) | 0.00587 | 0.3799 | −0.00206 | < 0.0001 | 0.00095 | 0.1006 |  |
| Protein intake (g/day) | 0.00401 | 0.5229 | −0.00047 | 0.3402 | 0.00037 | 0.4982 |  |
| Fat intake (g/day) | −0.00207 | 0.7741 | 0.00041 | 0.4638 | 0.00080 | 0.1968 |  |
| β, partial regression coefficient; BW, body weight.  The model included age, years of education, current smoking status, dietary restrictions, medical history, physical activity level, and body fat percentage as covariates. Macronutrient (outcomes) and ethanol consumption are standardized for 60 kg of BW. | | | | | | | |

Figure S5. Sensitivity analysis.

Differences in energy and macronutrient intakes between the *ALDH2* rs671 genotypes were tested in stratified cohorts by sex and by ethanol consumption levels. The general linear regression models included age, ethanol consumption amount, education years, current smoking status, medical history, dietary restrictions, and physical activity level as covariates. Error bars represent 95% confidence intervals. § Energy intake excluding energy from alcoholic beverages. * *p < 0.05*, ** *p < 0.01*, *** *p < 0.001*, and **** *p < 0.0001* between *ALDH2*1/*1* and *ALDH2*1/*2*.

| Table S9. Estimated energy and macronutrient intakes based on *ALDH2* rs671 polymorphism and ethanol consumption levels. | | | | | | | | | | |
| --- | --- | --- | --- | --- | --- | --- | --- | --- | --- | --- |
|  | | Non-drinkers | | | Moderate drinkers | | | Heavy drinkers | | |
|  | | *ALDH2*2*  (-) | (+) |  | *ALDH2*2*  (-) | (+) |  | *ALDH2*2*  (-) | (+) |  |
| Total energy intake (kcal/day) | | | | | | | | | | |
|  | Model I | 1708 | 1699 |  | 1691 | 1725 |  | 1759.9 | 1811.4 |  |
|  | *p* for difference | *0.3958* | |  | *0.0118* | |  | *0.0038* | |  |
|  | Model II | 1711 | 1698 |  | 1690 | 1727 |  | 1755.1 | 1826.9 |  |
|  | *p* for difference | *0.2349* | |  | *0.0053* | |  | *< 0.0001* | |  |
|  | Model III | 1711 | 1697 |  | 1691 | 1729 |  | 1755.5 | 1820.6 |  |
|  | *p* for difference | *0.1941* | |  | *0.0047* | |  | *0.0002* | |  |
| Energy intake excluding energy from alcoholic beverages (kcal/day) | | | | | | | | | | |
|  | Model I | 1707 | 1699 |  | 1646 | 1687 |  | 1531 | 1611 |  |
|  | *p* for difference | *0.435* | |  | *0.0026* | |  | *< 0.0001* | |  |
|  | Model II | 1710 | 1697 |  | 1647 | 1685 |  | 1533 | 1604 |  |
|  | *p* for difference | *0.235* | |  | *0.0053* | |  | *< 0.0001* | |  |
|  | Model III | 1710 | 1696 |  | 1648 | 1686 |  | 1534 | 1599 |  |
|  | *p* for difference | *0.1942* | |  | *0.0047* | |  | *0.0002* | |  |
| Carbohydrate intake (g/day) | | | | | | | | | | |
|  | Model I | 245 | 243 |  | 240 | 243 |  | 227 | 240 |  |
|  | *p* for difference | *0.375* | |  | *0.1221* | |  | *< 0.0001* | |  |
|  | Model II | 245 | 243 |  | 240 | 243 |  | 227 | 238 |  |
|  | *p* for difference | *0.2279* | |  | *0.2198* | |  | *0.0006* | |  |
|  | Model III | 245 | 243 |  | 240 | 243 |  | 227 | 237 |  |
|  | *p* for difference | *0.244* | |  | *0.1788* | |  | *0.0018* | |  |
| Protein intake (g/day) | | | | | | | | | | |
|  | Model I | 55.6 | 55.4 |  | 53.1 | 54.6 |  | 50.7 | 52.3 |  |
|  | *p* for difference | *0.774* | |  | *0.0005* | |  | *0.0054* | |  |
|  | Model II | 55.6 | 55.4 |  | 53 | 54.7 |  | 50.7 | 52.3 |  |
|  | *p* for difference | *0.6218* | |  | *0.0003* | |  | *0.0047* | |  |
|  | Model III | 55.7 | 55.4 |  | 53.1 | 54.7 |  | 50.7 | 52.2 |  |
|  | *p* for difference | *0.5802* | |  | *0.0004* | |  | *0.0086* | |  |
| Fat intake (g/day) | | | | | | | | | | |
|  | Model I | 46.1 | 45.8 |  | 42.3 | 44.3 |  | 38.3 | 39.4 |  |
|  | *p* for difference | *0.4369* | |  | *< 0.0001* | |  | *0.0196* | |  |
|  | Model II | 46.2 | 45.8 |  | 42.2 | 44.4 |  | 38.3 | 39.5 |  |
|  | *p* for difference | *0.3102* | |  | *< 0.0001* | |  | *0.0119* | |  |
|  | Model III | 46.4 | 45.8 |  | 42.4 | 44.4 |  | 38.3 | 39.4 |  |
|  | *p* for difference | *0.1689* | |  | *< 0.0001* | |  | *0.0215* | |  |
| Model I included age and sex as covariates; model II included covariates of model I and amount of ethanol consumption (continuous); model III included covariates of model II in addition to years of education, current smoking status, medical history, dietary restrictions, and physical activity level. Energy and macronutrient intakes are standardized for 60 kg of body weight. | | | | | | | | | | |
